# CRISPR-clear imaging of melanin-rich B16-derived solid tumors

**DOI:** 10.1038/s42003-023-04614-7

**Published:** 2023-04-04

**Authors:** Rajib Schubert, Taegeun Bae, Branko Simic, Sheena N. Smith, Seong-Ho Park, Gabriela Nagy-Davidescu, Viviana Gradinaru, Andreas Plückthun, Junho K. Hur

**Affiliations:** 1 Department of Biochemistry, University of Zürich, Zürich, Switzerland; 2 Division of Biology and Biological Engineering, California Institute of Technology, Pasadena, CA USA; 3 Department of Medicine, Graduate School, Kyung Hee University, Seoul, South Korea; 4 Department of Medicine, Major in Medical Genetics, Graduate School, Hanyang University, Seoul, South Korea; 5 Department of Genetics, College of Medicine, Hanyang University, Seoul, South Korea; 6 Department of Pathology, College of Medicine, Kyung Hee University, Seoul, South Korea; 7 Present Address: Research and early development, Roche Sequencing Solutions, Pleasanton, CA USA; 8 Present Address: College of Pharmacy, The Catholic University of Korea, Gyeonggi-do, South Korea; 9 Present Address: Vector BioPharma AG, Basel, Switzerland

**Keywords:** Genetic engineering, 3-D reconstruction, Confocal microscopy, Cancer models, Transcriptomics

## Abstract

Tissue clearing combined with deep imaging has emerged as a powerful technology to expand classical histological techniques. Current techniques have been optimized for imaging sparsely pigmented organs such as the mammalian brain. In contrast, melanin-rich pigmented tissue, of great interest in the investigation of melanomas, remains challenging. To address this challenge, we have developed a CRISPR-based gene editing approach that is easily incorporated into existing tissue-clearing workflows such the PACT clearing method. We term this method CRISPR-Clear. We demonstrate its applicability to highly melanin-rich B16-derived solid tumors, including one made transgenic for HER2, constituting one of very few syngeneic mouse tumors that can be used in immunocompetent models. We demonstrate the utility in detailed tumor characterization by staining for targeting antibodies and nanoparticles, as well as expressed fluorescent proteins. With CRISPR-Clear we have unprecedented access to optical interrogation in considerable portions of intact melanoma tissue for stained surface markers, expressed fluorescent proteins, of subcellular compartments, and of the vasculature.

## Abstract (web summary)

Tyrosinase CRISPR knock out in the B16 melanoma cell line eliminates melanin production, with the lack of pigment not affecting tumour engraftment or growth and allowing for 3D imaging after clearing by PACT.

## Introduction

Driven by research on the mammalian brain, various tissue-clearing methods have been established to enable 3D large-volume optical imaging^1–3^. A central feature in these tissue-clearing methods is homogenizing the refractive index and the removal of lipids in unpigmented tissues. However, a major limitation of this approach is that it is not easily applicable to tissues exhibiting melanin pigmentation, as melanin is a pigment known to cause strong light absorption and scattering and thus reduction in optical imaging quality^2,4^. This limitation is most likely due to melanin’s poor solubility in both lipid and water-based solvents^5,6^ and thus its resistance to extraction. Although several protocols exist for removing various naturally occurring pigments using either tissue-clearing protocols or various bleaching agents and acetone in combination with tissue clearing, the results of these efforts on melanin removal have shown limited success^6–8^. To address these limitations, Tikoo et al.^9^ recently applied a combined CRISPR editing of melanoma workflow in combination with a tissue expansion-based clearing termed CUBIC to a B16 mouse melanoma model, a highly melanin-rich solid tumor model^10–12^, which can also be used to investigate antibody and nanoparticle targeting. Here we evaluate an alternative clearing approach called PACT (Passive Clarity Technique) and expand on their integrated CRISPR-based gene editing with a tissue-clearing workflow in the B16 mouse melanoma model. Particularly, we also used a B16 tumor line that overexpresses HER2, which we used in a previously described fully-immunocompetent syngeneic C57BL/6 model which is tolerant to the transgenic marker^13^. We evaluate the utility of our approach in antibody staining and in nanoparticle imaging, which can inform but also constitute therapeutic intervention, at both cellular and subcellular resolution. We term our approach CRISPR-Clear.

## Results and discussion

### Melanin pigmentation in metastatic melanoma

Murine B16 melanoma is one of the most common syngeneic tumor models used to evaluate immune responses to immunotherapy and interactions among tumor cells, immune infiltrate and the tumor microenvironment^14^. Our goal was to generate a B16 variant that was amenable to deep 3D fluorescence microscopy with a full panel of immunofluorescent approaches: (1) visualization of encoded fluorescent protein, (2) immunohistochemistry (IHC) staining of a transgenic surface marker, (3) IHC staining of the vasculature with endogenous markers, (4) visualization of fluorescently labeled nanoparticles as a model therapeutic, and (5) conventional fluorescence staining, e.g., DAPI for cell nuclei. To address these five parameters, we used a previously described, poorly immunogenic sub-clone of B16 melanoma (D5) that stably expresses human epidermal growth factor receptor 2 (HER2) as a transgenic marker, called B16-D5-HER2^13^ (Fig. S1a). Furthermore, we generated a variant that additionally expresses stably integrated tdTomato fluorescent protein for assessment of endogenously encoded fluorescence, called B16-D5-HER2-TdTom (Fig. S1b, c). Our initial attempts to reduce pigmentation of B16 cells in vitro using hydroquinone, an inhibitor of the enzyme that catalyzes melanin biosynthesis^15^, tyrosinase, led to an incomplete loss of pigmentation and a loss of cell viability (Fig. S2); thus, the CRISPR knock-out approach was pursued.

### CRISPR editing of B16 cells

**Fig. 1:**
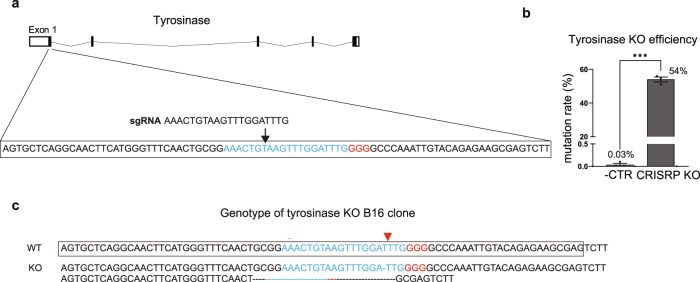
Melanin elimination in B16 cells via Cas9-sgRNA mediated genome editing of tyrosinase. **a** The tyrosinase target site for CRISPR genome editing. Sequence of the CRISPR target site at tyrosinase exon1. (Blue letters indicate spacer sequences of CRISPR-Cas9 and red letters indicate the PAM motif of CRISPR-Cas9 (5′-NGG-3′)). **b** Mutation rate of CRISPR-Cas9 introduced into B16 cells via lipofection. **c** Genotype of the tyrosinase-KO B16 clone used for imaging studies. Shown are the wild type (wt) reference sequence and the sequences of the two alleles of the tyrosinase-KO clone. One allele of the clone carries a 1-bp deletion, the other has a 46-bp deletion.

We prepared CRISPR-Cas9 and designed sgRNA for specific knock-out of the tyrosinase gene^16^ (Figs. 1a and S3a). In order to minimize off-target effects, the sgRNA sequence was selected such that any potential off-target site contained at least three base mismatches compared to the target sequence (Fig. S3b). We next delivered the CRISPR-Cas9 and tyrosinase-targeting sgRNA expression plasmids into B16 cells and assessed the efficacy of the CRISPR methods (Fig. 1b). We observed that the CRISPR procedure targeting tyrosinase resulted in mutation rates of ~54 %, and the mutated sequences consisted of small insertions and deletions (indels). After confirming the high efficacy, single cell clones of the CRISPR-treated B16 cells were individually assessed for tyrosinase knock-out (Fig. S3c). In one of the selected clones, we found that the two tyrosinase alleles of the genomic DNA contained 1-bp and 46-bp deletions, respectively (Fig. 1c). Both deletions in the tyrosinase DNA sequences are anticipated to induce frameshifting that results in loss of the tyrosinase activity.

We next sought to assess whether the tyrosinase-KO B16 clone contained mutations only at the targeted locus and was otherwise identical to their precursors. For this purpose, we searched for potential off-target mutations that could have been inadvertently introduced into the tyrosinase-KO cell clone. To this end, we analyzed the genomic DNA of the clone by targeted deep sequencing of the tyrosinase CRISPR target locus and 13 potential off-target loci that contained 3 mismatches compared to the target sequence (Fig. S3b, d). The sequencing analyses showed that the mutation rate of the on-target tyrosinase site was at least 99.5%, and all of the 13 off-target sites had mutation rates below detection error level of 0.5%, suggesting that the CRISPR based tyrosinase knockout did not lead to non-specific editing.

The results strongly suggested that the selected tyrosinase-KO clone contained a genomic background identical to the precursor cells, and we sought to assess the loss-of-function of tyrosinase in the clone. RT-qPCR was performed to verify the deletion of the tyrosinase gene, and for this purpose cDNA was generated from 1 μg of isolated total RNA and a reverse primer was designed with a 3′ end requiring the wt sequence for amplification. No amplification was obtained, showing unequivocally that the CRISPR approach for tyrosinase gene deletion was successful (Fig. S4a).

To confirm that the tyrosinase gene is also functionally deleted at the protein level, we performed Western blot analyses using lysates of both wt and tyrosinase-KO B16 cells stimulated with α-melanocyte-stimulating hormone (α-MSH) in cell culture. We concluded that we could not detect a tyrosinase band in the extracts of knocked-out B16 cells stimulated with α-MSH after 48 h and 72 h (Fig. S4b), while this band was detectable in wt B16 cells under the same conditions, when the cells reach >80% confluency, showing again that the CRISPR approach was successful. Finally, phenotypically, pellets from the melanin knock-out lacked black pigmentation compared to the wt and showed a brighter pinkish-red color, likely from the tdTomato fluorescent protein (Fig. S4c).

### CRISPR approach results in a melanin(–) B16 clone with otherwise virtually identical properties

We next investigated whether CRISPR genome editing might have introduced other changes, undetected by deep sequencing, to the B16 cells that might have altered their properties as a tumor model. To this end, we conducted transcriptome analyses of the tyrosinase-KO B16 cell line and the parental B16 cell line by RNA sequencing. The gene expression profiles of the KO and the parental B16 cells were overall very similar (Fig. S5a). We found that the expression levels of only four genes showed differences with potential statistical significance (Fig. S5b, c). However, the functional annotations of the four genes suggested that were not obviously related to cancer or melanin synthesis, and instead their expression may be rather related to cell cycle or culture conditions. Further analyses of the expression levels of genes related to cancer and the melanin synthesis pathway did not show any significant differences between the KO and parental B16 cell lines (Fig. S5d, e).

Finally, to ensure the melanin(–) cells conformed to the phenotypic characteristics observed by the melanin(+) cells at both the cell and tissue level, we analyzed both edited and non-edited cells at the single-cell level on the flow cytometer. Results from the forward and side scatter profiles show no measurable differences and suggest that the cell morphology is not affected and identical to the original B16-HER2 cells (Fig. S6a). Furthermore, expression levels of the transgenic marker, HER2, and the tdTomato fluorescent protein were similar to the parental cell line in Western blots (Fig. S6b), as was imaging from IHC of cultured and fixed cells for HER2 (Fig. S6c).

### In vivo analysis of CRISPR-Clear

Two murine models were used to assess tyrosinase-deleted B16 cells in vivo: (1) immunodeficient RAG1^–/–^ mice, which have no endogenous mature B or T cells and allow for investigation of the molecular architecture of the tumor microenvironment in the absence of an intact immune system, and (2) a previously described^13^ fully-immunocompetent syngeneic C57BL/6 model which is tolerant to the transgenic marker, HER2 (C57BL/6-Tg(HER2)). Bilateral tumors were engrafted subcutaneously on the flanks of each mouse (i.e., melanin(+) on the right flank and melanin(–) on the left flank) to control for intrinsic variabilities among individual animals that would influence tumor development and outgrowth. Tumors were grown to for 25-40 days to volumes of 1700 mm^3^, and we observed no measurable differences between the size or morphology of B16 wt and KO-derived tumors grown on either RAG1^–/–^ (Fig. S7a, b) or C57BL/6-Tg(HER2) (Fig. S7c, d) background at extraction. For detailed imaging experiments, we focused on the syngeneic C57BL/6-Tg(HER2) model.

In summary, these results confirm that a melanoma cell line was created that merely differs from B16 in the knock-out of tyrosinase in both alleles and otherwise has retained the B16 phenotype, examined at the DNA, RNA and protein level, as well as by cell morphology and tumor development.

### Optical analysis of CRISPR-Clear B16 tumors

**Fig. 2:**
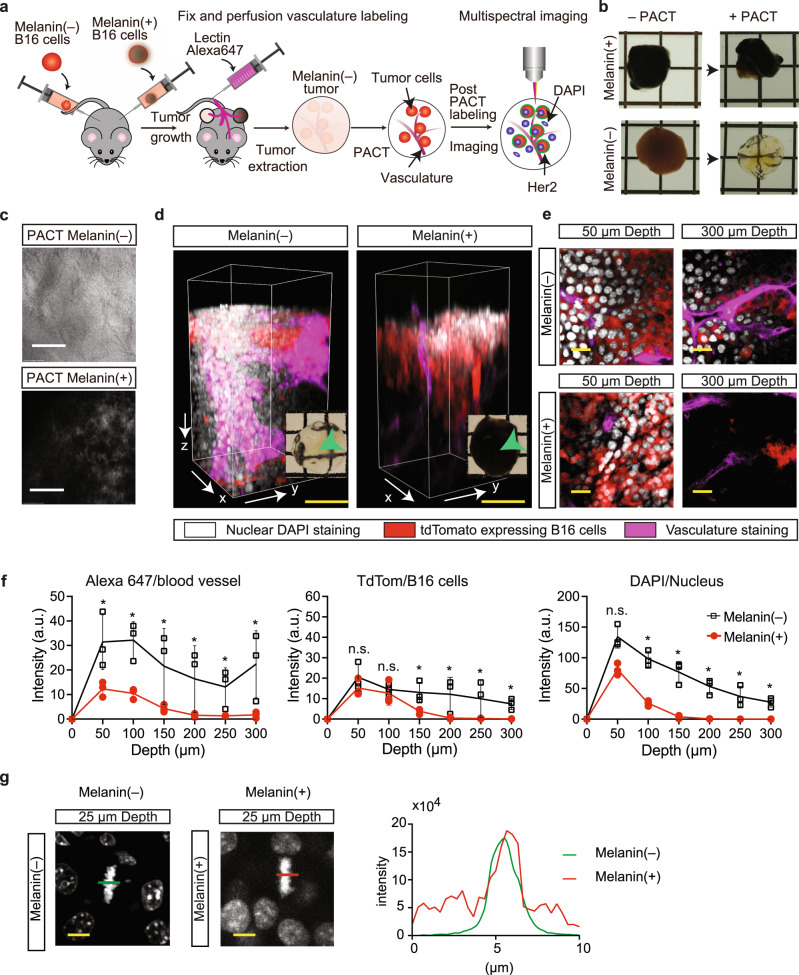
Imaging of intact melanin(+/−) tumors. **a** Schematic overview of tumor generation, PACT rendering, and labeling of intact adult mouse tumors with and without tyrosinase-edited melanin B16 cells. **b** Optical transparency comparison of 1 mm^3^ PACT-cleared intact adult mouse tumors with and without tyrosinase-edited melanin B16 cells before and after PACT clearing, grid lines are 10 × 10 mm. **c** DIC imaging of surfaces of intact tumors with and without tyrosinase-edited melanin B16 cells before and after PACT clearing, scale bar 500 µm. **d** Three-dimensional view of confocal imaging at the center of intact tumors with the 25x objective, 300 µm depth. White represents nuclear DAPI staining, red represents tdTomato-expressing B16 cells, and purple represents vasculature straining. *Arrowhead*, snapshot of imaged tumor, grid lines are 10 mm × 10 mm. Scale bar 100 μm (yellow). **e** Single slices from imaged volume in **d**. **f** Signal intensity in arbitrary units (A.U.) versus depth from dye-labeled blood vessels to determine scattering lengths in labeled tumor regions shown in **d**. *p-value <0.01, using 2-tailed Student’s *t* test. **g** *Left*, high-resolution imaging using a 63x objective of nuclei stained with DAPI in intact tumors with and without melanin; *right*, quantification of background compared to signal noise in (A.U.), using ratio plots obtained from fluorescence intensity profiles along the green and red lines in **g** across mitotic cells in metaphase. Scale bar 10 μm (yellow).

To assess the in vivo optical properties of intact tissues subjected to CRISPR-Clear, we excised a set of bilateral tumors when volumes reached up to 1700 mm^3^ (day 25–40) from C57BL/6-Tg(HER2) mice and performed PACT clearing on them (Fig. 2a). We observed a dramatic difference in optical translucence when tumors grown from melanin(–) cells were compared to those grown from melanin(+) cells; however, no measurable morphological difference was found between the two tumors that were subjected to PACT (Fig. 2b). Additionally, we noticed striking differences when the tumors were observed under the differential interference (DIC) microscope (Fig. 2c). Most notably, we noted a complete absence of any dark spots in the melanin(–) tumors, but also a striking difference in surface features.

We next compared melanin(–) and melanin(+) B16 tumors (Fig. 2a, d) in detail. We imaged blood vessels that had been labeled by *i.v*. injection of DyLight 649 in the tail vein prior to sacrifice and fixation, while B16 cells were imaged by their tdTomato fluorescence and their nuclei with DAPI. To ensure that vasculature imaging reflected the variation in the optical properties when comparing melanin(–) and melanin(+) tumors, and was not due to artifacts from faulty labeling, we controlled labelling by performing slice imaging on other tissues from the same animal (Fig. S8). Using the Zeiss-880 confocal microscope with the multimersion 25x objective with glycerol mounting media (Fig. 2d, e, Supplementary video 1), we noticed a 10- to 30-fold increase in signal intensities for the melanin(–) tumor at 300 µm depths, consistent across all fluorophores, and labelling agents (Fig. 2f). We observed a similar trend in our large-volume imaging with a depth 1045 µm below the surface of the tumor, and in intact tumor imaging (Fig. S9, Supplementary video 2). These differences between melanin(–) and melanin(+) tumors were found independently of whether they were grown in a RAG1^–/–^ or a C57BL/6 background (Fig. S10).

In a last set of experiments, we assessed the optical properties of subcellular elements with two additional labeling experiments: high resolution imaging of nuclei (Supplementary Video 3) and 300 nm diameter fluorescent nanoparticle localization in 500 µm thick tissue slices (Supplementary Video 4). For the first strategy we imaged 1 mm tissue slices on melanin(+)/(–) tumor tissue with the 63x objective using glycerol immersion (Fig. 2g, Supplementary video 3). Our imaging and analyses revealed a dense mesh of punctate structures, most likely nuclear bodies^17^, in the nuclei of melanin(+) tumors not present in the melanin(–) tumors, and we observed a higher signal-to-background ratio at depths of up to 50 µm in the latter. This shows that the CRISPR-edited cells dramatically improved subcellular imaging.

### Analysis of antibody penetration depth and nanoparticles in B16-tumor-bearing mice

**Fig. 3:**
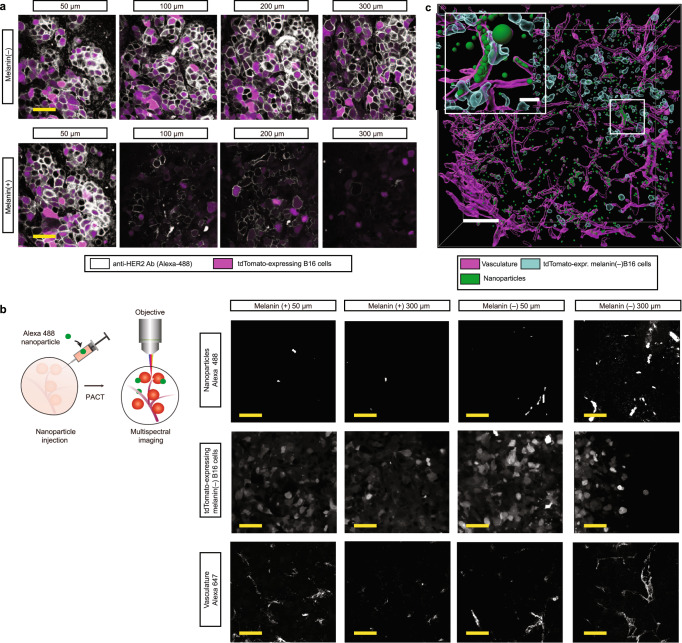
Multimodal labeling characterization. **a** Comparison of antibody labeling of intact melanin (+/–) tumors. HER2 is detected with an anti-HER2 antibody and stained with an Alexa-488 labeled secondary antibody (shown in white), tdTomato-expressing B16 cells (shown in purple), imaged at increasing depths. Images were acquired using a 25x objective. Scale bar 150 μm (yellow). **b** Overview of workflow for 3D imaging, localization and visualization of nanoparticles in melanin(–) tumors. The method consists of 2 modular steps: (1) multi-dye vessel-staining (highlighted in Fig. 2a) and injection of nanoparticles in tissue, and (2) clearing for high imaging quality using 3D confocal microscopy. For clarity, separate channels are shown for nanoparticles (Alexa 488), tdTomato-expressing melanin(–) B16 melanoma cells and vasculature (Alexa 647). Scale bar 300 μm. A 10x/0.45 objective was used for imaging. **c** 3D visualization of digital reconstruction of nanoparticles at 500 µm depth of melanin(–) tumors cleared with PACT^18^. Purple is vasculature, cyan are reconstructed tdTomato-expressing melanin(–) B16 melanoma cells, and green represents nanoparticles. Scale bar 300 μm. White box insert on the left is a blow-up of the box on the right. Scale bar 20 μm for insert. A 10x/0.45 objective was used for imaging.

Of particular interest in histology applications for tissue clearing is antibody labeling of desired molecular targets. In previous studies using PACT-based tissue clearing, improved antibody penetrance compared to non-cleared samples was shown^3,18,19^. To assess CRISPR-Clear’s combability with antibody staining we assessed the expression levels of the transgenic marker HER2 using IHC staining of HER2 in intact tumors at depths ranging from 50 µm to 300 µm. Using a 25x objective we imaged both melanin(+) and melanin(–) tumors. Consistent with results on imaging endogenous fluorescence markers we observed consistent labeling of HER2 throughout the imaging volume on the melanin(–) tumors but a strong drop-off in labeling in melanin(+) tumors (Fig. 3a). The drop-off in labeling is most likely due to optical dampening by melanin itself as seen in the previous figures (Fig. 2 and Fig. S10). Our results thus demonstrate that the CRISPR-Clear approach is a viable technique compatible with robust antibody labeling.

The syngeneic B16 murine melanoma model is the gold standard for pre-clinical studies in immuno-oncology and therapeutic intervention studies using nano-drugs for cancer biology^20,21^. As a demonstration of CRISPR-Clear’s applicability in accessing drug delivery, we injected nanoparticles to mimic nanoparticle-based therapies within the tumor microenvironment in immunodeficient RAG1^–/–^ mice. Using a 10x objective and digital reconstruction of the optical images we were able to observe not only the nanoparticles themselves but also the subcellular localization of them in the vasculature (Fig. 3b, c and Supplementary video 4) in B16 melanin(–) tumors.

## Conclusions

In summary, we show the applicability of CRISPR-Clear to completely abolish melanin formation and thus enable high resolution deep tissue imaging with minimal perturbation to the biological system of the widely used murine B16 melanoma, one of the very few syngeneic mouse tumor models. We conclude that the tyrosinase-KO, melanin(–) tumors show no other significant differences to the progenitor B16 melanoma, neither at the morphological, cancer pathway or transcriptomics level.

Beyond providing access to the optical interrogation of strongly pigmented tissues in conjunction with clearing methods, CRISPR-Clear also has the potential to be expanded to other clearing techniques, as used in whole-animal imaging and future in vivo workflows with further improved clearing technologies.

## Methods

### B16 melanin(+) tdTomato cell line generation

The generation and characterization of a lentivirus encoding tdTomato has been described previously^22^. The B16-D5-HER2 stable cell line was a generous gift from Louis Weiner (Georgetown University)^13,23^. Cell lines were cultured in DMEM Dulbecco’s modified Eagle’s medium (DMEM; Sigma) supplemented with 10% (v/v) fetal calf serum (FCS; Amimed) and 1% (v/v) penicillin/streptomycin solution (5000 U/mL; Thermo Fisher) at 37 °C, 5% CO_2_ and >90% humidity.

### CRISPR-mediated B16 melanin(–) tdTomato cell line generation

Melanin(+) B16 cells were transfected with CRISPR-Cas9 targeting tyrosinase, the cells were diluted to single cells cultured in 96-well plates. Each cell clone was individually genotyped to identify tyrosinase knock-out clones.

### Cell culture and transfection

B16 cells were cultured in DMEM containing 10% FCS and 1% (v/v) penicillin/streptomycin solution. For CRISPR-Cas9 delivery, cells were seeded in 24-well plates at 70–80% confluency and were transfected using Lipofectamine 2000 (Invitrogen) as described previously^24^. Briefly, the cells were transfected with a Cas9 expression plasmid (500 ng) and sgRNA expression plasmids (500 ng). The transfected cells were cultured for 72 h, and the genomic DNA was isolated with a DNeasy Blood & Tissue Kit (Qiagen) for subsequent analyses. The cells were both confirmed to be free of mycoplasma contamination.

### Mutation analyses at on-target and off-target homologous sites and knockout clone selection

The B16 cells that were transfected with CRISPR-Cas9 were assessed for mutation rate as described previously^16^. To this end, we conducted nested PCR (700–1000 bp amplicons) followed by targeted deep sequencing (primer information is included in Table S1). We used Cas-OFFinder (http://www.rgenome.net) to find potential off-target sites that differed from on-target sequences by up to 3 bp^25^. The genomic regions of the on-target site and potential off-target sites were amplified by PCR using Phusion polymerase (Thermo Fisher Scientific) to assess the mutation rates by CRISPR-Cas9. The regions were amplified by nested-PCR (two rounds) to add the Illumina adaptors (P5 and P7). The amplified PCR samples were purified and subjected to paired-end read deep sequencing using an Illumina iSeq 100 instrument. The sequencing data were analyzed by Cas-Analyzer^26^. We obtained the frequencies of insertions and deletions located 3 bp upstream of the PAM (around the CRISPR-Cas9 cleavage site), that were considered mutations induced by CRISPR-Cas9. After confirming the mutation rate, the cells were diluted to single-cell culture in 96-well format. Each clonal cell culture was individually genotyped to identify a tyrosinase-KO clone to be used for imaging experiments.

### RT-PCR

RT-PCR was carried out on the B16 tyrosinase-KO clone and the B16 wt cell line to verify the presence of the frameshifting mutations. The RT-qPCR easy-spin™ [DNA free] Total RNA Extraction Kit (iNtRON) was utilized for RNA extraction. RNA extraction was performed according to the manufacturer’s instructions. cDNA synthesis was performed using 1 μg of isolated total RNA using the High-Capacity cDNA Reverse Transcription Kit (Applied Biosystems) according to the manufacturer’s instructions. For quantitative RT-PCR (qRT-PCR), cDNA was put in each well of a Hard-Shell® 96-Well PCR Plates (Bio-Rad). THUNDERBIRD™ Next SYBR® qPCR Mix(SYBR green, TOYOBO) provided fluorescence signals to quantify target cDNA amounts. Relative mRNA expressions were analyzed by the ΔΔCt method. Reactions were run on a CFX Connect Real-Time PCR System (Bio-Rad).

### Western blot

For verification of successful deletion of the tyrosinase gene, the B16 wt and B16 ΔTyr cells from T150 ml flasks were seeded in concentration of 1 × 10^5^cells/ml onto 9 cm cell culture dishes (Cat. No. 20101, SPL Life Sciences) using DMEM containing 1% penicillin/streptomycin (Cat. No. 15070063, ThermoFisher) supplemented with 10% FCS (Cat. No. 2-01F10-I, BioConcept). Cells were placed in a 37 °C/5% CO_2_ incubator, and 24 h post-seeding, the medium was removed from all the plates, and replaced with 10 ml fresh DMEM containing 50 nM α-MSH (Cat. No. M4135-1MG, Sigma Aldrich). Cells were further incubated at 37 °C/5% CO_2_ and after 24 h, the plates were harvested using cell culture lysis reagent (Cat. No. 1531, Promega), supplemented with 58 mM DTT and a cocktail of protease inhibitors (pefabloc, leupeptin, and pepstatin-A, final dilution 1:500), with plates being incubated at 4 °C for 1 h on a rotating platform before cells were scraped using cell scrapers (Cat. No. 800020, Bioswisstec). Then the lysates were transferred into 1.5-ml tubes, vortexed thoroughly, centrifuged at maximal speed for 20 min at 4 °C and the supernatants were collected and stored at −20 °C. For the remaining plates, the old medium was replenished with the fresh medium containing fresh α-MSH, harvesting plates after 48 h and plates after 72 h, with medium/MSH replenished daily. Untreated plates were harvested once cells reached ≥80% confluency, which was 48 h after seeding.

Protein concentrations were determined with the BCA assay (Cat. No. 23252, Thermo Fisher). Forty μg of protein lysates prepared with Laemmli sample buffer (Cat. No. 1610747, Bio-Rad) were boiled at 99 °C for 10 min, cooled to room temperature, centrifuged and loaded onto 4–20% Mini-Protean TGX Stain-free gels (Cat. No. 4568095, Bio-Rad), and ran in TGX premixed electrophoresis buffer (Cat. No. 1610772, Bio-Rad) at 200 V. Proteins were transferred to a LF PVDF membrane (Cat. No. 10026934, Bio-Rad) that was previously activated with 100% methanol and subsequently washed with water, repeating the activation and rinsing step three times, and finally incubated in transfer buffer (Cat. No. 10026938, Bio-Rad) for 15 min. The transfer was performed in Trans-Blot® Turbo™ Transfer System (Cat. No. 1704150EDU, Bio-Rad). Following the transfer, the membrane was incubated in blocking buffer (Cat. No. B6429, Sigma Aldrich) for 30 min at room temperature on a rotating platform, and then incubated with the primary antibodies (Rabbit anti-mouse tyrosinase pAb, C-terminus, Cat. No. LS-C46797, LifeSpan BioSciences, 1:250 in antibody dilution buffer-blocking buffer with TBS-T; mouse anti-GAPDH, Cat. No. sc-32233, Santa Cruz, 1:3000 in antibody dilution buffer-blocking buffer with TBS-T) at 4 °C overnight on an orbital shaker. Two antibodies claimed by the vendors to recognize mouse tyrosinase in Western blot failed, however: (i) TYR Antibody, MaxPab® Rabbit Polyclonal, Abnova, Catalog # H00007299-D01P and (ii) Tyrosinase Monoclonal Antibody (T311), Invitrogen, Catalog # MA5-14177. The next day the membranes were washed 4 × 20 min with TBS-T, and goat anti-rabbit IgG conjugated with alkaline phosphatase (Cat. No. A3687, Sigma Aldrich) or goat anti-mouse IgG conjugated with alkaline phosphatase (Cat. No. A3562, Sigma Aldrich) was used as the second antibody in 1:10,000 in antibody dilution buffer-blocking buffer with TBS-T for 1 h at room temperature on the rotating platform. The membranes were washed 4 × 20 min with TBS-T and developed using the CDP-Star Chemiluminescent substrate (Cat. No. C0712, Sigma Aldrich) on a Vilber Fusion Fx Spectra system.

Expression of HER2 and tdTomato was tested in experiments without α-MSH stimulation, with the following primary antibodies (1:1000 dilution): Anti-HER2/ErbB2 antibody (Cell Signaling, #2165), anti-tdTomato antibody (KeraFAST, EST203), using goat anti-rabbit IgG (H + L) secondary antibody-HRP (Thermo Fisher Scientific, #31460, and HRP goat anti-rat IgG antibody (Vector Laboratories, PI-9400).

### RNA isolation

For measuring potential differences in gene expression between B16 wt and B16 trosinase-KO cells, total RNA was isolated using Trizol reagent (Invitrogen). RNA quality was assessed with an Agilent 2100 bioanalyzer (Agilent Technologies, Amstelveen, The Netherlands), and RNA quantification was performed using an ND-2000 Spectrophotometer (Thermo Inc, DE, USA).

### RNA library preparation, sequencing and analysis

Libraries were prepared from total RNA using the NEBNext Ultra II Directional RNA-Seq Kit (New England BioLabs, Inc, UK). The isolation of mRNA was performed using the Poly(A) RNA Selection Kit (Lexogen, Inc, Austria). The isolated mRNAs were used for cDNA synthesis and shearing, following the manufacturer’s instructions. Indexing was performed using the Illumina indexes 1-12. The enrichment step was carried out by using PCR. Subsequently, libraries were checked using the Agilent 2100 bioanalyzer (DNA High Sensitivity Kit) to evaluate the mean fragment size. Quantification was performed using the library quantification kit using a StepOne Real-Time PCR System (Life Technologies, Inc, USA). High-throughput sequencing was performed as paired-end 100 sequencing using HiSeq X10 (Illumina, Inc, USA). The read files (FASTQ) were mapped to the mouse genome by hisat2, and the counts per genes were calculated by the htseq-count software. Next, the differential expression profiles of the genes were analyzed by DESeq2^27^. The plots were generated using the plotMA and ggmaplots functions in the R package. The scheme of the genetic pathway for melanin biogenesis was prepared based on previous studies^28,29^.

### Flow cytometry and cell pellet images

Cells were grown to 80–100% confluency, washed with Dulbecco’s PBS (DPBS), and detached from culture plates by the addition of trypsin-EDTA solution (Sigma T3924) and incubation at 37 °C for 3–5 min. The trypsinized cells were transferred into complete media, centrifuged at 300 *×* *g* for 3 min at 4 °C, and washed in cold PBS containing 1% BSA (bovine serum albumin; PBS/BSA). Images of cell pellets were taken following aspiration of the PBS wash solution. For assessment of tdTomato expression and scattering properties of the cell line, cells were read directly without staining. For assessment of HER2 expression levels, cells were added to wells of V-bottom flow cytometry plates (Greiner 651201), centrifuged at 300 × *g* for 3 min, and supernatants were aspirated. The cells were resuspended in 50 μL of an anti-ErbB2 (HER-2), FITC-conjugated monoclonal antibody (clone 2G11; eBioscience Cat. No. BMS120FI) at a 1:100 dilution in PBS/BSA and incubated in plates at 4 °C for 1 h with gentle orbital shaking. Cells were washed twice with 250 μL PBS/BSA, then resuspended in 100 μL PBS/BSA per well before reading. Sample readings were acquired without cell fixation on an LSRII Fortessa flow cytometer using a high-throughput sampler and FACSDiva software (Becton Dickinson), and data were analyzed with FCS Express 5 (De Novo Software).

### Hydroquinone treatment

Cells were seeded on 15 cm plates and grown to 80–100% confluency. Once confluent, hydroquinone (Fluka, Cat. No. 53960) was added to plates to final concentrations ranging from 6.25–50 μM from a 10 mM stock made in DPBS (and incubated at 37 °C, 5% CO_2_ for 24-hours). Cells were imaged on a light microscope at various magnifications, then cells were washed with Dulbecco’s PBS, detached from culture plates by the addition of trypsin-EDTA solution (Sigma T3924) and incubation at 37 °C for 3–5 min. The trypsinized cells were transferred into complete media, centrifuged at 300 *×* *g* for 3 min at 4 °C, and washed in cold PBS containing 1% BSA (bovine serum albumin; PBS/BSA). Images of cell pellets were taken following aspiration of the PBS wash solution.

### Animal experiments

The Rag1^−/−^ and C57BL/6 hmHER2 transgenic mouse strains were generous gifts from Manfred Kopf (ETH Zürich) and Louis Weiner (Georgetown University)^13,23^, respectively. Animals were confirmed to be free of pathogens and housed according to Federation of European Laboratory Animal Associations (FELASA) recommendations under BSL2 conditions. Mice were housed in individually ventilated type III plastic cages (425 × 266 × 150 mm, floor area 820 cm^2^, 2–5 animals per cage) with sterile dust-free wooden bedding (80–90 g per cage; Schill AG, Muttenz, Switzerland), a red translucent plastic mouse house and paper tissues (2 per cage) for nesting. Mice were provided a pelleted mouse diet (Kliba No. 3431, Provimi Kliba, Kaiseraugst, Switzerland) and sterilized drinking water *ad libitum*. The room was kept at 21 ±  1 °C, 50 ± 5% relative humidity with a 12 h light/12 h dark cycle (40 lux artificial light from 07.00 to 19.00 h). Cages were individually ventilated with filtered air (15 complete air changed per hour, 50 Pa, HEPA H 14 filter, Vokes-Air, Uster, Switzerland). All animal experimentation was approved by the Cantonal Veterinary Office (Zurich, Switzerland) permit Nr. 237/2017, and carried out in accordance with Swiss animal protection laws and the European Convention for the protection of vertebrate animals used for experimental and other scientific purposes (Council of Europe no. 123 Strasbourg 1985). Prior to engraftment of mice with tumors, cell lines were verified to be pathogen-free to comprehensive PCR testing for FELASA mouse pathogens (IDEXX Laboratories). Cells were washed three times in ice-cold DPBS and passed through a 40 μm cell strainer (SPL Life Sciences Ltd) prior to subcutaneous injection of 100 μL volume in either the left or right flank of female C56BL/6-hmHER2^TG^ (*n* = 9) and RAG1^−/−^ (*n* = 9) of 4–5 months of age. For comparison of the wt and tyrosinase-KO cell lines, tumors were grown on the same mouse on contralateral flanks until they reached euthanasia criteria. Mice were given a lethal dose of 100 mg/kg ketamine and 10 mg/kg xylazine prior to transcardial perfusion fixation as previously described^30^. Tumors and livers were excised, placed into histology cassettes and submerged in 4% PFA (Electron Microscopy Sciences, cat. no. 15710-S) for 8-12 h. Tissues were then washed with PBS three times (12 h each), and then stored in PBS (Electron Microscopy Sciences, cat. No.19242-40) containing 0.2% sodium azide.

### Vasculature labeling

Prior to perfusion fixation (approximately 10–15 min), mice were injected intravenously with 100 μl DyLight 649 labeled *Lycopersicon esculentum* Lectin (Vector Laboratories Cat. No. DL-1178).

### Nanoparticle experiments

B16 tumors were grown on the Rag1^–/–^ mice as described above. One hour before sacrifice, 50 μl of fluorescent-green silica nanoparticles (PSI-G0.2, Kisker Biotech GmbH & Co. KG, Steinfurt, Germany) were intratumorally injected. Blood vessels staining, animal euthanasia and tissue preservations were performed as described above.

### PACT Clearing and refractive index homogenization

PACT clearing was performed as previously described^18^. Briefly, four percent paraformaldehyde (PFA)-fixed tissue of varying sizes were incubated at 4 °C overnight in 4% acrylamide in PBS, supplemented with 0.25% photo-initiator 2,2’-azobis[2-(2-imidazolin-2-yl)propane] dihydrochloride (VA-044, Wako Chemicals USA). Samples were then degassed with nitrogen for 1–5 min and then incubated for 2–3 h at 37 °C to initiate tissue-hydrogel hybridization. After removing excess hydrogel via brief PBS washes, tissue-hydrogel matrices were transferred into 50 ml conical tubes containing 8% SDS (Sigma cat. No. 05030) in 0.1 M PBS (pH 7.5), and were incubated for 2–5 days at 37 °C with shaking and accessed to transparency before removing them and washing them in PBS. All staining and mounting steps were conducted at room temperature with gentle shaking. Samples were incubated in RIMS as previously described^18^ until transparent, followed by mounting in fresh RIMS.

### Imaging of PACT-cleared samples with a DSLR camera

Imaging of the samples before and after clearing was done using a Sony DSLR Alpha350 camera, 14.2 megapixel with the N50 3.5-5.6/18-70 Macro and wide angle zoom lens.

### Fluorescence Microscopy

Cleared tissue samples were incubated in RIMS solution for one day. The samples were then mounted in the respective solutions using 7.0 mm or 3.0 mm spacers (iSpacer, SunJin Lab) with cover glasses. The mounted samples were stored at room temperature and shielded from light prior to imaging. Fluorescent images and initial sample visualization were performed on a conventional confocal microscope (Zeiss LSM 880) with either a Plan-Apochromat 5X/0.45 M27 objective (working distance 6.0 mm), 10 × 0.45 N.A. Plan-Apochromat objective, LD LCI Plan-Apochromat 25X/0.8 Imm Corr DIC M27 multi-immersion objective (working distance 0.57 mm) and 63x/1.3 glycerol DIC M27 (working distance 0.30 mm). After imaging, samples were embedded in RIMS at room temperature and protected from light for storage.

### Quantification Methods

3D image visualization, image quantification and image reconstructions were performed using Imaris version 9.5 imaging software (Bitplane) as previously described^19,20^. Fluorescence measurements were analyzed using the open source software FIJI. Statistical analysis was performed using Prism (version 6.0b for Mac OSX; GraphPad Software).

### Statistics and Reproducibility

Statistical significance was evaluated by a two-tailed Student’s *t*-test where indicated. In other experiments, the number of samples is indicated in the respective figure caption. Error bars denote S.D. The types of replicates are defined in the figure captions.

### PACT Immunohistochemistry and small molecule labeling

Immunostaining was done as previously described^18^. Briefly, PACT-processed samples were washed in PBS and then transferred to buffer containing small-molecule dyes or primary antibodies, followed by fluorescently conjugated secondary antibody in PBS containing 2% normal donkey serum, 0.1% Triton X-100 and 0.01% sodium azide) for 3–7 days or with small-molecule dyes for 1–3 days. Antibody or small-molecule dye solutions were replaced daily. Unbound antibody was removed via PBS washes as before, and then samples were incubated with secondary antibodies (Fab fragment secondary antibodies were preferred, diluted 1:200–400) for 2–5 days, then washed for 1 day in PBS prior to incubation in imaging media (refractive index matching solution, RIMS). The primary antibodies used for passive staining were rabbit anti-HER2 IgG (Polyclonal Antibody, ThermoFisher PA5-14635). An AlexaFluor 488-conjugated donkey anti-rabbit IgG (Jackson ImmunoResearch, West Grove, PA) was used for the secondary antibody staining (Fig. S6), followed by washing in PBS for one hour. The tissues were then placed in RIMS solution for 4 h prior to imaging. All steps were performed at room temperature.

### Reporting summary

Further information on research design is available in the Nature Portfolio Reporting Summary linked to this article.

## Supplementary information

Supplementary Information

Description of Additional Supplementary Files

Supplementary Video 1. Imaging of melanin(–) B16 tumors

Supplementary Video 2. Imaging of melanin(–) B16 tumors with a depth 1045 µm below the surface of the tumor.

Supplementary Video 3. High resolution imaging of nuclei and fluorescent nanoparticle localization

Supplementary Video 4. Analysis of nanoparticles in B16-tumor-bearing mice.

Supplementary Data 1

Reporting Summary

## Data Availability

The sequencing data are available at the NCBI Sequence Read Archive (SRA, http://www.ncbi.nih.gov/sra) under the accession number PRJNA910290. All other data are available from the corresponding author (or other sources, as applicable) on reasonable request. Associated raw data for Figs. 1b, 2f, S4a, S5e, S7b and S7d can be found as Supplementary Data, and uncropped western blots are shown in Fig. S11.
