# Supplementary material for: CRISPR-clear imaging of melanin-rich B16-derived solid tumors
Source: Commun Biol. 2023 Apr 4;6:370. doi: 10.1038/s42003-023-04614-7 (PMC10073193; doi:10.1038/s42003-023-04614-7)
Supplement: Supplementary file 1 — Supplementary Information [file 42003_2023_4614_MOESM1_ESM.pdf]

Supplementary Information

CRISPR-clear imaging of melanin-rich B16-derived solid tumors

Rajib Schubert[1,2,7†*], Taegeun Bae[3,8†], Branko Simic[1,9†], Sheena N. Smith[1,9†], Seong-Ho Park[4], Gabriela Nagy-Davidescu[1], Viviana Gradinaru[2], Andreas Plückthun[1*] and Junho K. Hur[5,6*]

[1]Department of Biochemistry, University of Zürich, Zürich, Switzerland

[2]Division of Biology and Biological Engineering, California Institute of Technology, Pasadena, CA, USA

[3] Department of Medicine, Graduate School, Kyung Hee University, Seoul, South Korea

[4] Department of Medicine, Major in Medical Genetics, Graduate School, Hanyang University, Seoul, South Korea

[5] Department of Genetics, College of Medicine, Hanyang University, Seoul, South Korea

[6] Department of Pathology, College of Medicine, Kyung Hee University, Seoul, South Korea

[7] **Present address:** Research and early development, Roche Sequencing Solutions, Pleasanton, CA, USA

[8] **Present address:** College of Pharmacy, The Catholic University of Korea, Gyeonggi-do, South Korea

[9] **Present address:** Vector BioPharma AG, Basel, Switzerland

[†]These authors contributed equally to this work

[*]**Correspondence**: Rajib Schubert (rac0820@gmail.com), Andreas Plückthun (plueckthun@bioc.uzh.ch) and Junho Hur (juhur@hanyang.ac.kr)

Supplementary figures:

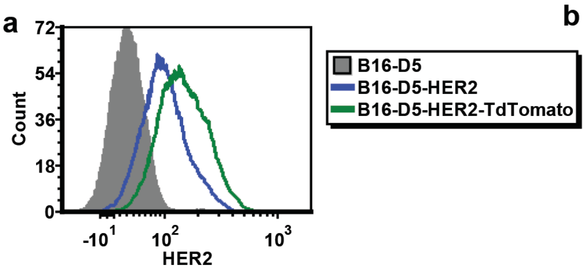

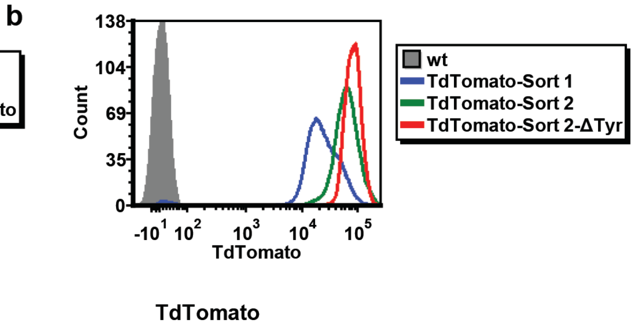

Figure S1: Generation of a tdTomato-positive B16-D5-HER2 cell line (**a)** Relative HER2 surface levels of the parent B16-D5-HER2 cell line and the B16-D5-HER2-tdTomato line generated with a tdTomato-encoding lentivirus via flow cytometry of cells stained with an anti-HER2 antibody. (**b**) Flow cytometry histograms showing tdTomato expression following FACS progression for selection of a tdTomato-positive population from lentivirus transformed B16-D5-HER2, from which the CRISPR-Clear variant was made (i.e., tdTomato-Sort 2-ΔTyr.

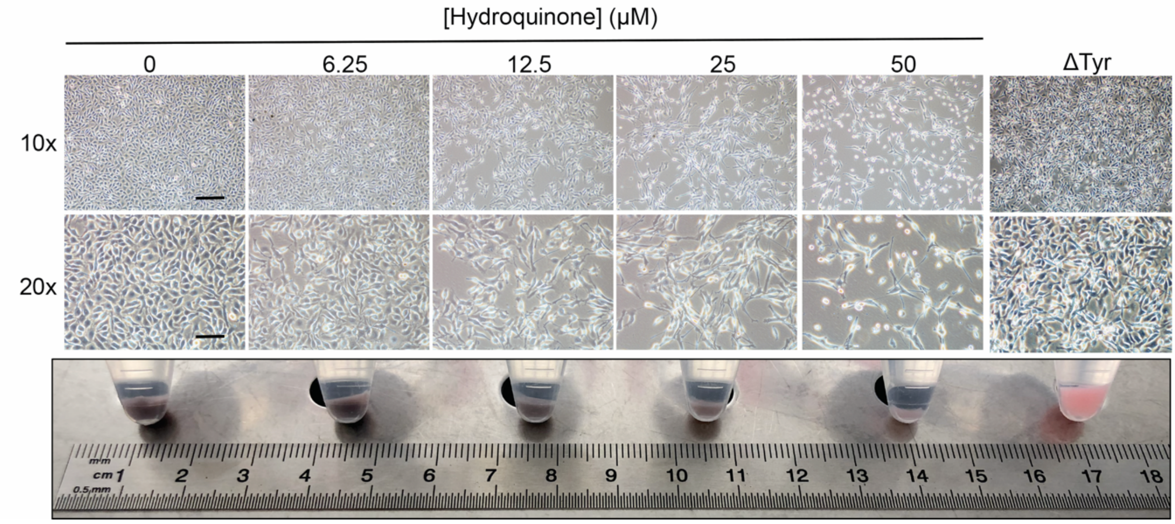

Figure S2: *Top*, Bright-field images (10x and 20x) of B16-D5-HER2-tdTomato grown for 24 h in the presence of hydroquinone in comparison to the tyrosinase-KO cell line (ΔTyr). *Bottom*, corresponding images of cell pellets. Scale bars: 10x, 100 μm; 20x, 50 μm.

a

sgRNA design for Tyrosinase KO with minimal off-target sites

Target	Sequence (5'-X20-NGG-3')	mismatches to on-target site *			
		0	1	2	3
Tyrosinase	AAACTGTAAGTTTGGATTTGGGG	1	0	0	13

b

Potential Off-target Sites (with 3 bp mismatches) - show matches with wt seq

Target	Sequence (5'-X20-NGG-3')	Chromosome	Position	Direction	Mismatches
**ON**	**AAACTGTAAGTTTGGATTTGGGG**	**chr7**	**87493025**	**-**	**0**
OT1	AAAtTtTAAGTTaGGATTTGAGG	chr3	41702527	-	3
OT2	AAACTGTgtGTTTGGATgTGAGG	chr7	42417217	+	3
OT3	AtACTGTAAGgTTaGATTTGTGG	chr4	14138070	+	3
OT4	cAACTGTAgGgTTGGATTTGTGG	chr4	51266568	-	3
OT5	AAtCTGTgAGTTTGGAgTTGAGG	chr1	125856398	+	3
OT6	AtACTGaAAGTTTGGAcTTGGGG	chr19	24426164	+	3
OT7	AAACaGTAAGTTTGtgTTTGTGG	chr15	32170570	-	3
OT8	AAAagGTAAtTTTGGATTTGAGG	chr10	100841863	+	3
OT9	AAACTGTAAGTcTGGtTTTtGGG	chr14	64427426	+	3
OT10	AAACTGgAAGTcTGGATaTGTGG	chr9	56057009	+	3
OT11	AAACTGTAtGTgTGtATTTGGGG	chrX	136080330	-	3
OT12	AAAaTGTgAGTTTGGtTTTGTGG	chr18	27242774	-	3
OT13	AAACTGTAAGccTGGATTgGAGG	chr18	62594928	-	3

c

Tyrosinase KO single cell clone analysis

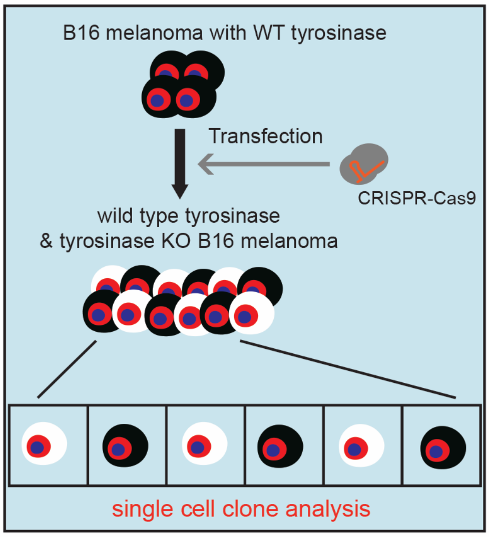

d

Mutation analysis of Tyrosinase KO clone
at potential off-target sites (3 bp mismatches)

Target	WT indel	TYR KO indel
**ON**	**0.2**	**99.5**
OT1	0	0
OT2	0	0
OT3	0.1	0
OT4	0.1	0.1
OT5	0	0
OT6	0.1	0.1
OT7	0	0
OT8	0	0
OT9	0.2	0.2
OT10	0	0
OT11	0	0
OT12	0.2	0.2
OT13	0	0

Figure S3: Generation of tyrosinase knock-out cells and off-target analyses. a, Computational design of CRISPR-Cas9 sgRNA for targeted knock-out of tyrosinase gene in B16 melanoma cell line. Shown are the numbers of potential off-target loci with a given number of mismatches within the mouse genome. The target site is selected to avoid potential off-

targets up to 2 mismatches compared to the on-target sequence (0 mismatches). The selected target site has 13 potential off-target sites with 3 mismatches. Red letters indicate the PAM motif of CRISPR-Cas9 (5'-NGG-3'). **b,** The DNA sequence, chromosome number, position, direction and the number of mismatches for the on-target and the 13 potential off-target sites are indicated. Small letters indicate mismatches compared to the on-target sequence, and red letters indicate the PAM motif of CRISPR-Cas9. **c,** Scheme of selecting single cell tyrosinase knock-out clones. After wt B16 melanoma were transfected with CRISPR-Cas9 targeting tyrosinase, the cells were diluted to single-cell culture in 96 well plates. Each single-cell clone was individually genotyped to select a tyrosinase knock-out clonal cell line. **d,** Off-target analyses of a tyrosinase knock-out B16 clone. Shown are the genome editing efficiencies estimated by qPCR at the on-target and at 13 potential off-target sites with 3 mismatches. For the selected knock-out clone, the mutation rates including insertions and deletions were 99.5% for the on-target and below 0.2% for 13 potential off-targets. In the parental B16 cell line, the mutation rates were below 0.2% for all tested on- and off-target sites.

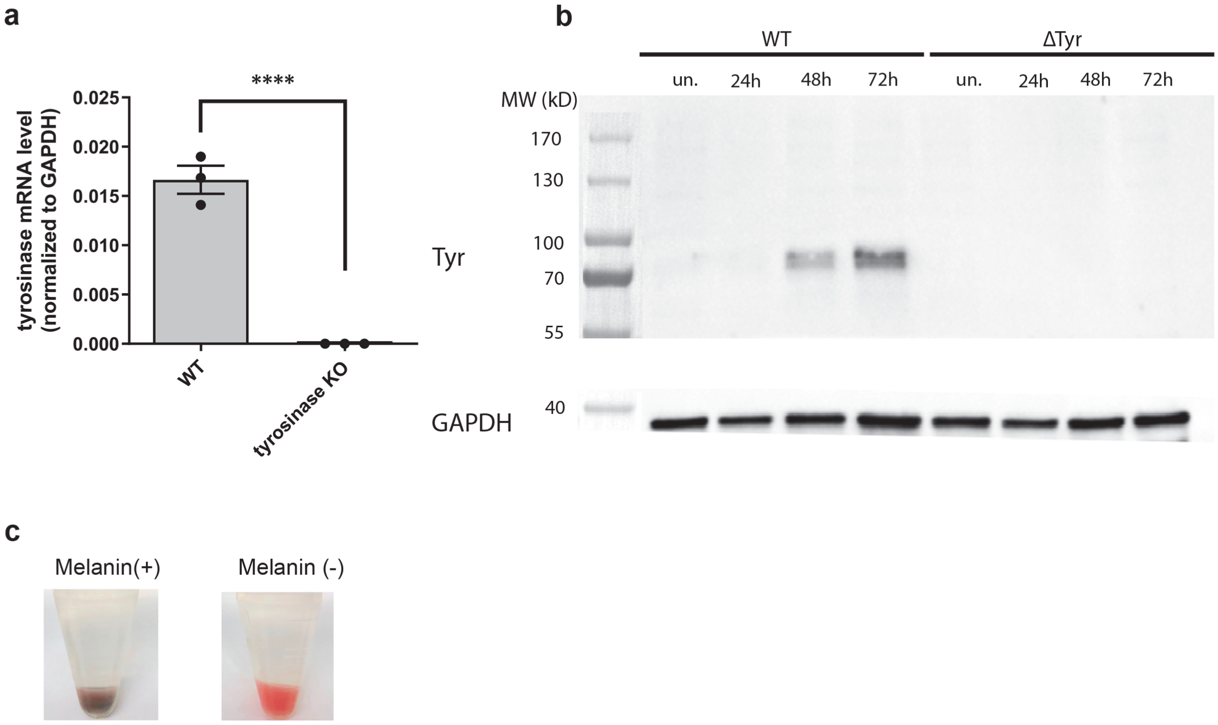

Figure S4: Phenotypic analysis of melanin(+/–) cells. a, RT -PCR from cDNA of wild type-B16 (WT) and tyrosinase-KO B16 cells. Statistical significance was evaluated by a two-tailed Student's t-test (**** p-value <0.001). **b,** Western blot analysis of protein extracts from wild type B16 (WT) and tyrosinase KO B16 cells (ΔTyr), stimulated with 50 nM α-MSH for 24, 48, and 72 hours, un. = unstimulated. **c**, corresponding images of cell pellets.

a

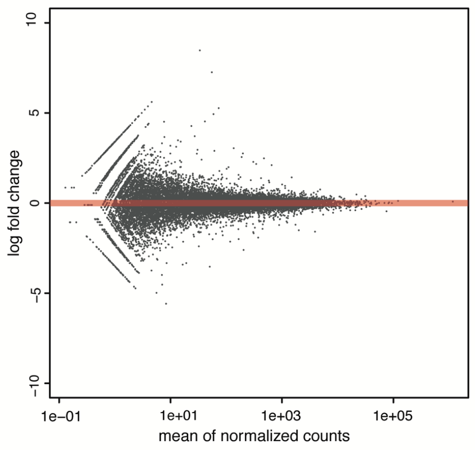

b

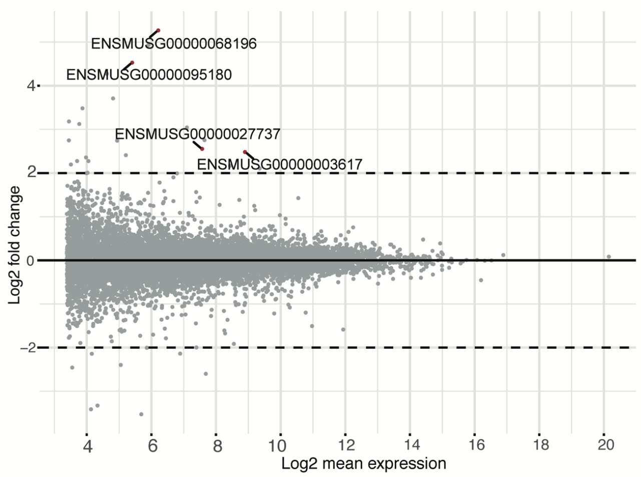

c

	Gene	Description	Gene ID	padj	Pathway/Function
1	Col8a1	Collagen, type VIII, alpha 1	ENSMUSG00000068196	3.67E-11	Extracellular matrix organization
2	Cp	Ceruloplasmin	ENSMUSG00000003617	8.73E-09	SLC-mediated transmembrane transport
3	Rhox5	Reproductive homeobox 5	ENSMUSG00000095180	5.58E-08	DNA binding
4	Slc7a11	Solute carrier family 7 (cationic amino acid transporter, y+ system), member 11	ENSMUSG00000027737	5.83E-04	SLC-mediated transmembrane transport

d

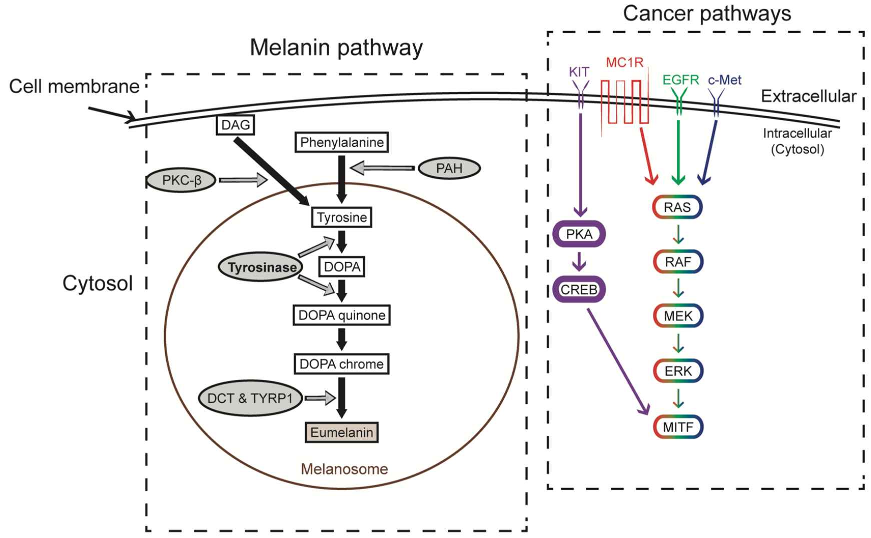

e

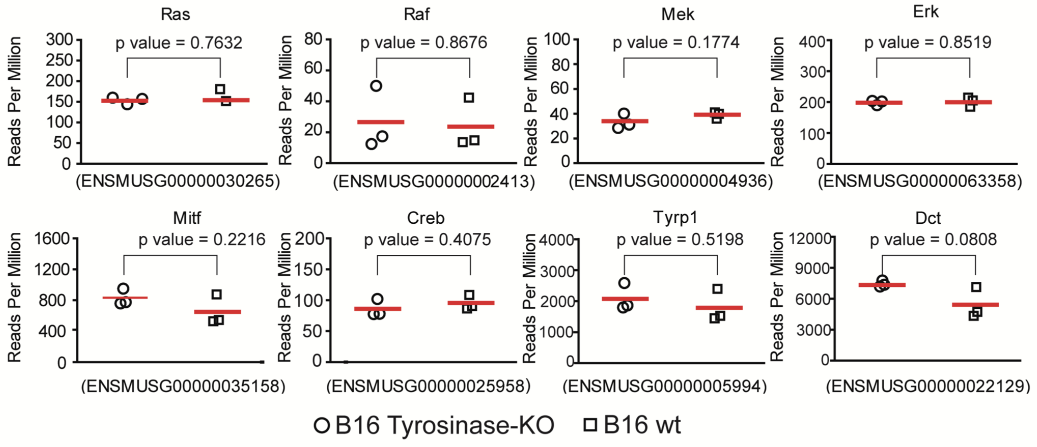

Figure S5: Comparative analyses of RNA expressions of melanin (+/–) cells. a, Differential expression analysis based on RNA-seq of wild-type B16 and of the tyrosinase knock-out cell line. **b,** Shown are the only four genes with potentially statistically different expression levels between the wild-type and the tyrosinase knock-out cell line (adjusted p-value below 0.05 and expression level differences larger than 4-fold). **c,** Descriptions of the four genes identified in the RNA-seq analyses. **d,** A scheme of melanogenesis pathway. Tyrosinase and other enzymes are shown that are involved in biosynthesis of melanin, and the melanogenic pathways are regulated by EGFR and other signaling pathways. **e,** RNA-seq analysis for comparison of expression levels of genes in the melanogenesis pathway. All differences are non-significant ($p > 0.05$).

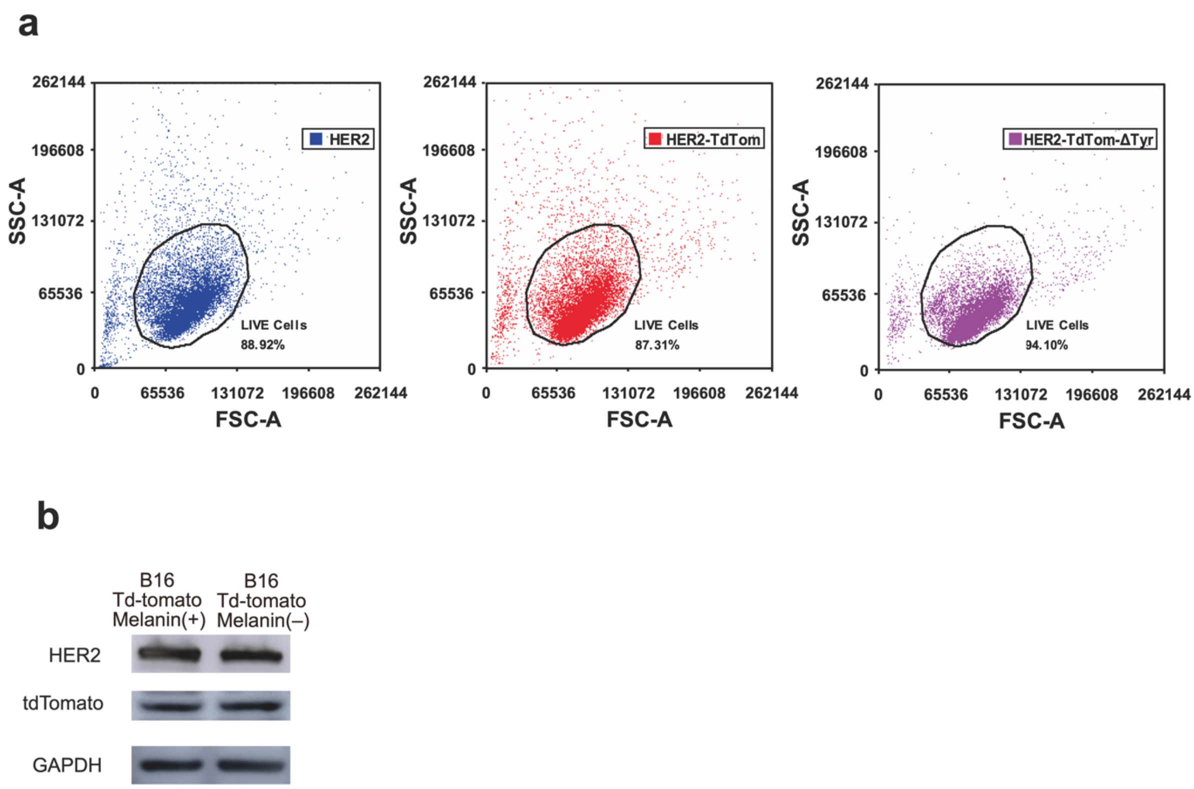

Figure S6: Validation of indistinguishable characteristics of B16-HER cells, additionally expressing tdTomato and carrying a tyrosinase knock-out. **a,** Forward and side scatter of cultured original B16-D5-HER2 cells (termed *HER2* for simplicity), the derived stably expressing tdTomato line (*HER2-TdTom*) and the tyrosinase knock-out cells derived from that (*HER2-TdTom-ΔTyr*). For each population, 8-10,000 events gated on live cell populations are shown. **b,** Western blot analyses of HER2 and tdTomato expression, with GAPDH loading control, in wild-type and tyrosinase knock-out cells.

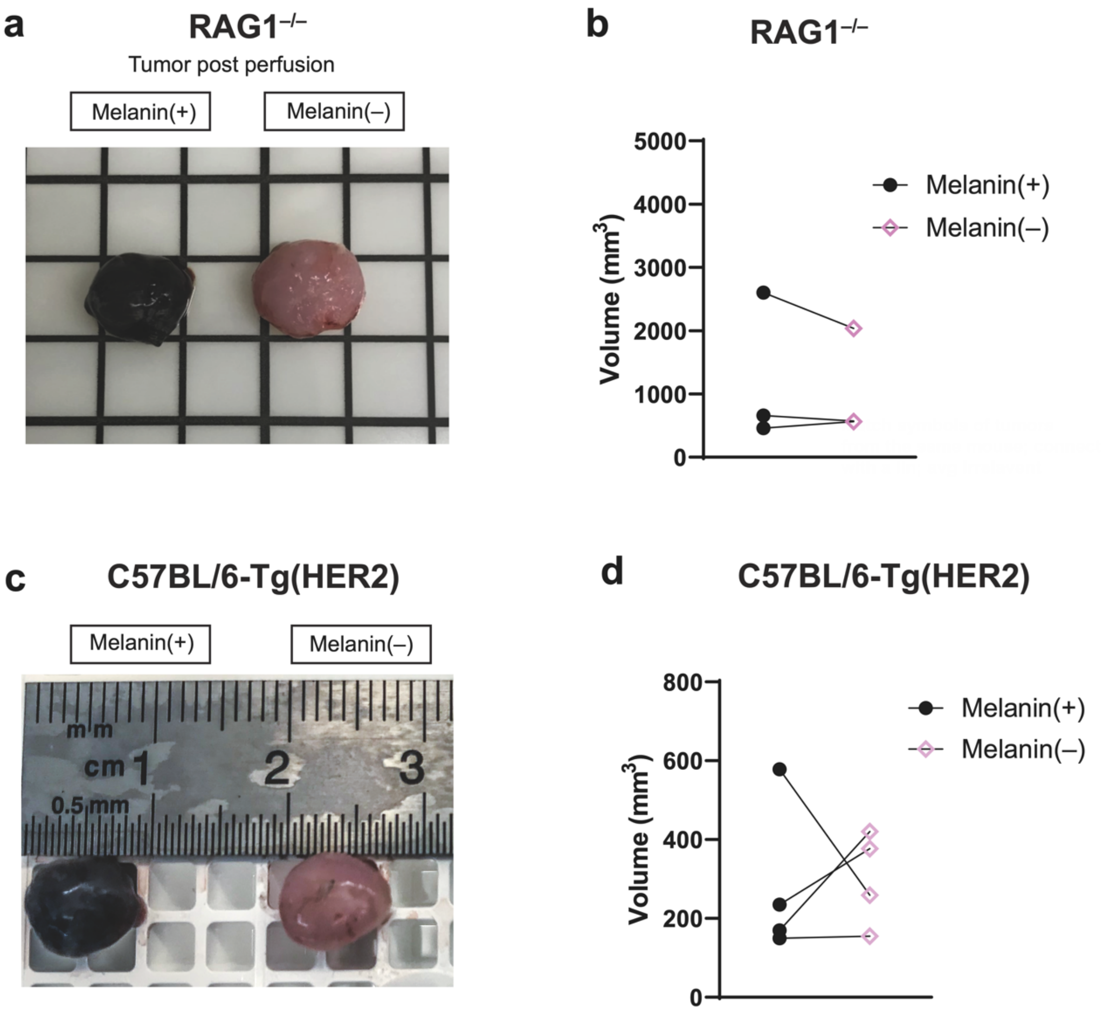

Figure S7: Representative tumor images and quantification of tumor volumes. a, tumors grown on the flanks of immunodeficient RAG1$^{-/-}$ and harvested after 25-40 days after B16 inoculation. grid size: 10x10 mm. **b**, quantification of tumor size from 3 different mice [tumor volume = $(\text{width})^2 \times \text{length}/2$]; connecting lines represents tumors belonging to the same animal. **c**, tumors grown on the flanks of fully-immunocompetent syngeneic C57BL/6-Tg(HER2) mice and harvested after 25-40 days after B16 inoculation; the ruler placed above indicates the tumor size. **d**, quantification of tumor size from 3 different mice [tumor volume = $(\text{width})^2 \times \text{length}/2$]; connecting lines represents tumors belonging to the same animal.

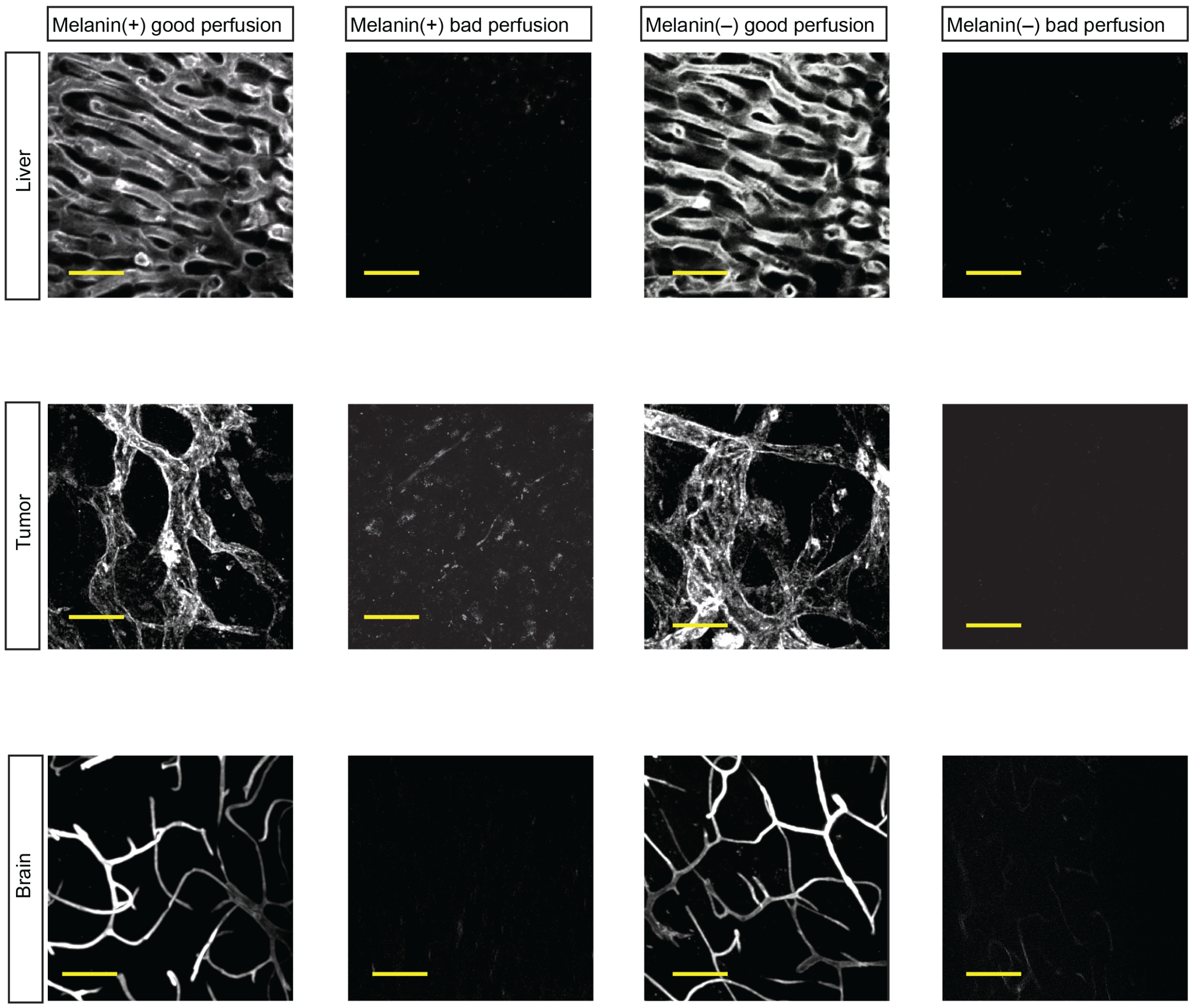

Figure S8. Vasculature imaging controls of melanin (+/-) tissue. Representative slice images of 3 different tissues showing vasculature staining of tissue. Only tissues showing good vasculature definition were considered for imaging analysis. Scale bar 100 µm.

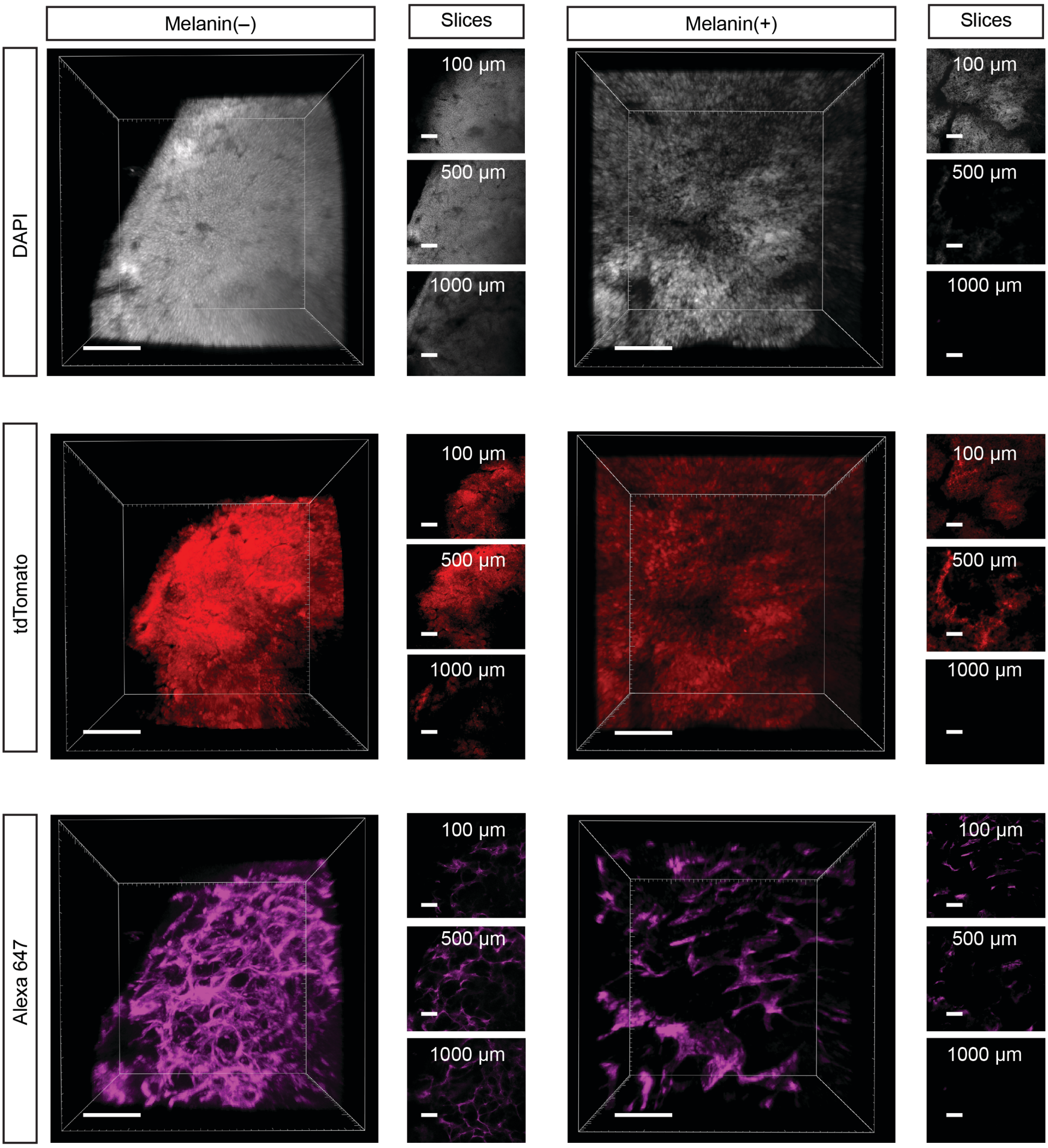

Figure S9. Deep tissue imaging comparison of intact tumors without and with formed melanin. Left, 3D visualization of tumors, visualizing three constituents, B16 cells (tdTomato), nuclei (DAPI), and blood vessels (Alexa 647-labeled lectin) imaged for about 1 mm depth into the center of the tumor. Image stack was taken with 10 μm axial steps. Right, slice images take n of corresponding 3D visualizations at 3 varying depths. Large pictures, scale bar 2 mm; small pictures, scale bar 500 μm.

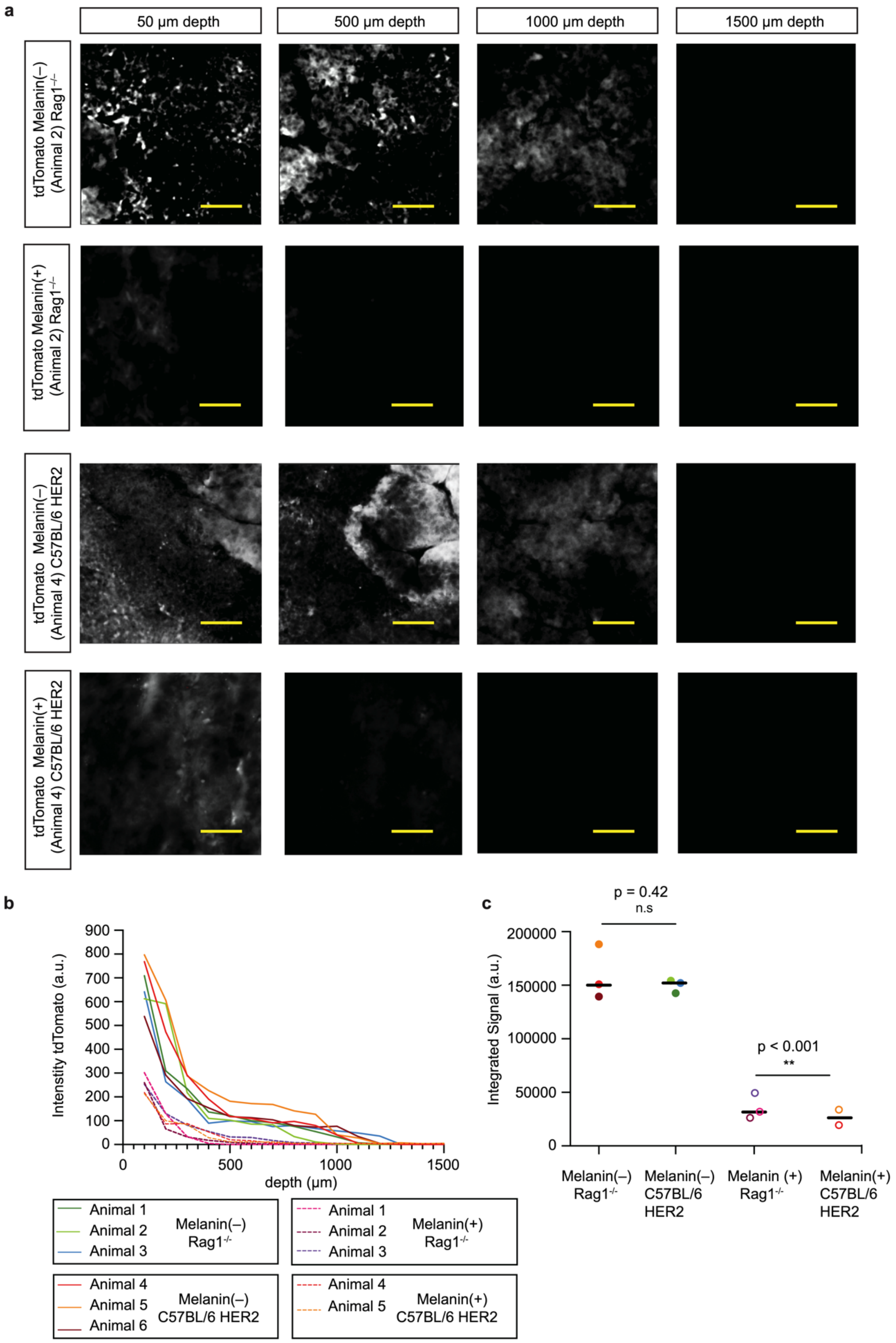

Figure S10. Endogenous tdTomato fluorescence comparison of intact tumors without and with formed melanin in two different backgrounds. a, Representative slice images of

endogenous tdTomato expression at varying depths of all the tumor types used in this study using a Zeiss Plan-Apochromat 10x/0.45 objective. Scale bar 100 µm. **b,** Total sum intensity of tdTomato signal in A.U across each slice image acquired from 50 µm to 1500 µm depth with 2 µm stack spacing from all tumor samples used in this study. **c,** The integrated tdTomato fluorescence intensity (area under the curve) in arbitrary units (A.U.) of panel **b**. N.S is not significant and ** p-value <0.001) using 2-tailed Student's t test.

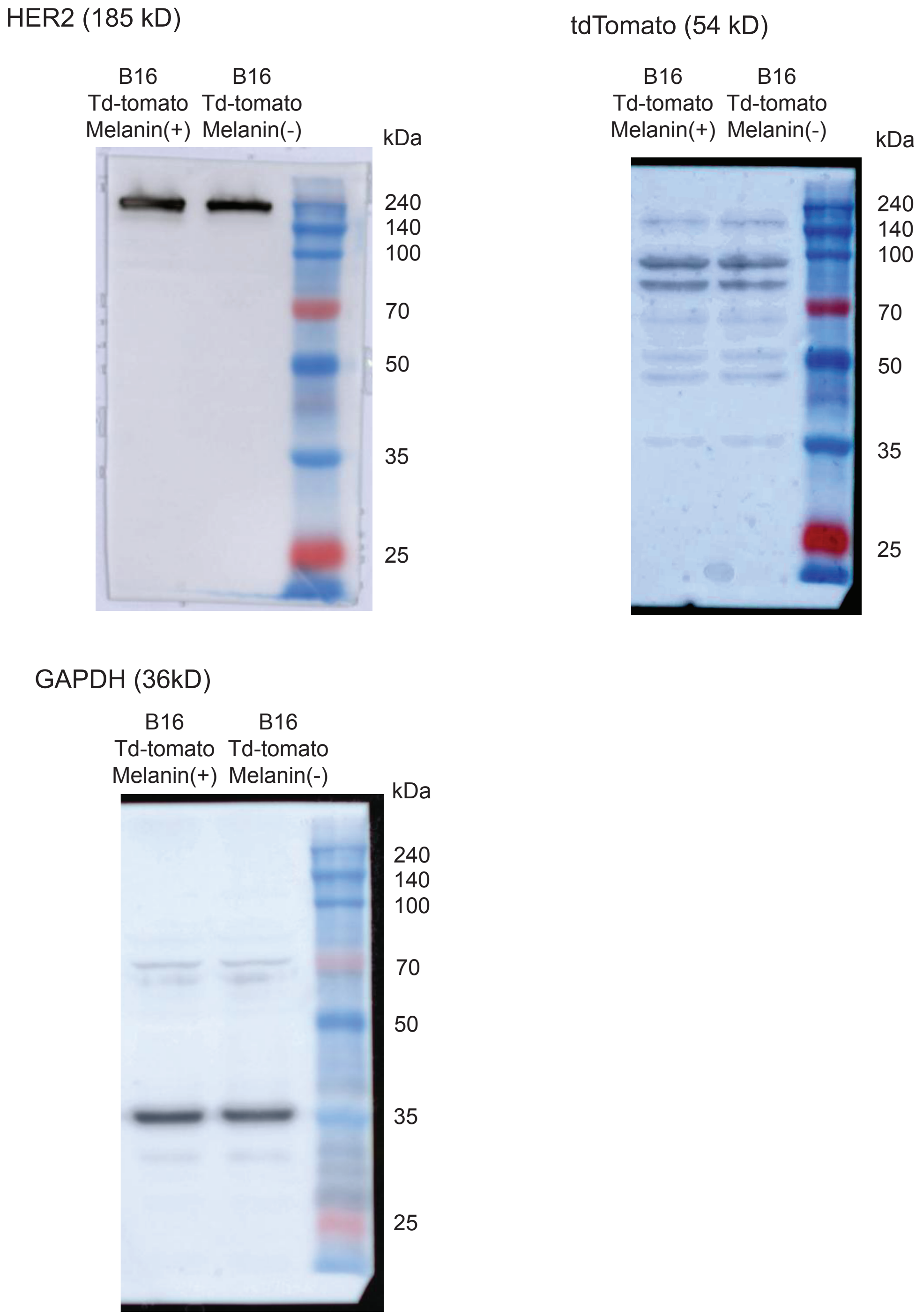

Figure S11: Uncropped western blot analyses of HER2 and tdTomato expression, with GAPDH loading control, related to Supplementary Figure S6b. Note that the contrast of the whole tdTomato blot was enhanced before cropping.

Table S1. DNA oligo and PCR Primer list

#	Name	Sequence
1	Cpf1_tyr_F	agatGATTTGGGGGCCCAAATTGTACA
2	Cpf1_tyr_R	aaaaTGTACAATTTGGGCCCCCAAATC
1	Cas9_tyr_F	caccgAAACTGTAAGTTTGGATTTG
2	Cas9_tyr_R	aaacCAAATCCAAACTTACAGTTTc
3	tyr_1st PCR_F	ACATGTGATAGTCACTCCAGGGGT
4	tyr_1st PCR_R	TGGGGATGACATAGACTGAGCTGA
5	tyr_2nd PCR_F	ACACTCTTTCCCTACACGACGCTCTTCCGATCTTCCTTCTGTCCAGTGCACCAT
6	tyr_2nd PCR_R	GTGACTGGAGTTCAGACGTGTGCTCTTCCGATCTAGCTGATAGTATGTTTTGCTAAAGTGAGGT
7	OT1_1st_F	ccttcaaatcagcacaaggaa
8	OT1_1st_R	atgcACCTAGGATGGATAGC
9	OT1_2nd_F	ACACTCTTTCCCTACACGACGCTCTTCCGATCTcagaggaccttcctagcattt
10	OT1_2nd_R	GTGACTGGAGTTCAGACGTGTGCTCTTCCGATCTcctctcttcccagttaactctt
11	OT2_1st_F	ttccaactccaggtttgct
12	OT2_1st_R	actgatgcccgacaaagt
13	OT2_2nd_F	ACACTCTTTCCCTACACGACGCTCTTCCGATCTagcttgtgggatgggttt
14	OT2_2nd_R	GTGACTGGAGTTCAGACGTGTGCTCTTCCGATCTGCAGCTATAAGCCCTGTACT
15	OT3_1st_F	ggtgcacttgcatttgagata
16	OT3_1st_R	agtaagagaaggagaagtttacca
17	OT3_2nd_F	ACACTCTTTCCCTACACGACGCTCTTCCGATCTtttgggttggctgatgtgc
18	OT3_2nd_R	GTGACTGGAGTTCAGACGTGTGCTCTTCCGATCTcccagactactatacccagcaaa
19	OT4_1st_F	gtaccaccagagcttgtatct
20	OT4_1st_R	gcttgtgtcttgtgtctcttac
21	OT4_2nd_F	ACACTCTTTCCCTACACGACGCTCTTCCGATCTccctaagagaccatagatgtcag
22	OT4_2nd_R	GTGACTGGAGTTCAGACGTGTGCTCTTCCGATCTgtgtgtgtgtctgtgtgattt
23	OT5_1st_F	TGTGCTCTGTCATCTCTCTTT
24	OT5_1st_R	TCCACCATCACATCCTTCTC
25	OT5_2nd_F	ACACTCTTTCCCTACACGACGCTCTTCCGATCTGTGGTAGGTTTCCAGGTAGTAG
26	OT5_2nd_R	GTGACTGGAGTTCAGACGTGTGCTCTTCCGATCTTCATCTATCACCACCACATCC
27	OT6_1st_F	CCACGACACTCCTATTGTGA
28	OT6_1st_R	GGCATGTTGGTTGGGTAAC
29	OT6_2nd_F	ACACTCTTTCCCTACACGACGCTCTTCCGATCTGCATGCTTAGTGTGCGTATG
30	OT6_2nd_R	GTGACTGGAGTTCAGACGTGTGCTCTTCCGATCTAAGCATCCTTAAGTGCTTCAAA

31	OT7_1st_F	acatcacactgtgtcctcaa
32	OT7_1st_R	TACATGGAAGGCAGAAGAGG
33	OT7_2nd_F	ACACTCTTTCCCTACACGACGCTCTTCCGATCTATCCAGCCTATGGCTCTAATG
34	OT7_2nd_R	GTGACTGGAGTTCAGACGTGTGCTCTTCCGATCTTTGGGTCACTGGCATGAATA
35	OT8_1st_F	tggctagtgttgaggtcttt
36	OT8_1st_R	GCCTCATATGTTACTTGCTTCC
37	OT8_2nd_F	ACACTCTTTCCCTACACGACGCTCTTCCGATCTAGTCATGAGAGAAATACCAAGACTA
38	OT8_2nd_R	GTGACTGGAGTTCAGACGTGTGCTCTTCCGATCTTTGTGAATCTGCAATGCTTTCT
39	OT9_1st_F	CTTCTCTTGTCCGTGGGATAC
40	OT9_1st_R	GATCTCAGCCGAGCCATATT
41	OT9_2nd_F	ACACTCTTTCCCTACACGACGCTCTTCCGATCTACGCTTCTCTTTGCTTTCTATG
42	OT9_2nd_R	GTGACTGGAGTTCAGACGTGTGCTCTTCCGATCTtcaaggtcatcctgtgctac
43	O10_1st_F	AGGTCTTGAGAAGGGACATAAA
44	OT10_1st_R	tcccgctAAAGGAAGTGAAT
45	OT10_2nd_F	ACACTCTTTCCCTACACGACGCTCTTCCGATCTttgccttcagctacataggg
46	OT10_2nd_R	GTGACTGGAGTTCAGACGTGTGCTCTTCCGATCTACAGTCCTGAAATAGGGACAAA
47	O11_1st_F	CACAGGGAGGGAAGAAGAAA
48	OT11_1st_R	GGGTTAGGAGGAGTTGTGTT
49	OT11_2nd_F	ACACTCTTTCCCTACACGACGCTCTTCCGATCTAAGACCTTACCTACAGCAGAC
50	OT11_2nd_R	GTGACTGGAGTTCAGACGTGTGCTCTTCCGATCTGAGCCCACATGCTAGATCA
51	OT12_1st_F	ctcaaaggaccagcaaacatc
52	OT12_1st_R	cctttcacgctgaggtagt
53	OT12_2nd_F	ACACTCTTTCCCTACACGACGCTCTTCCGATCTacaccagactttacaccagag
54	OT12_2nd_R	GTGACTGGAGTTCAGACGTGTGCTCTTCCGATCTagggcatagtttccgtcttt
55	OT13_1st_F	TGGCCATTTCTTTGCCTTAC
56	OT13_1st_R	CCCAGGATTTGCTCTGTTTG
57	OT13_2nd_F	ACACTCTTTCCCTACACGACGCTCTTCCGATCTgagcacatggctaggagaaa
58	OT13_2nd_R	GTGACTGGAGTTCAGACGTGTGCTCTTCCGATCTTCTTACTCGCCTCCTGAGTAT
59	mouse_GAPDH_F	CATCACTGCCACCCAGAAGACTG
60	mouse_GAPDH_R	ATGCCAGTGAGCTTCCCGTTCAG
61	Tyrosinase_F	ATAATAGGACCTGCCAGTGCTC
62	Tyrosinase_R	GTACAATTTGGGCCCCCAAA

DNA oligos 1 and 2 were used to construct the sgRNA expression plasmid for CRISPR knock-out of tyrosinase. Primers 3 to 6 were utilized for targeted deep sequencing to analyze the mutation rates at the on-target CRISPR genome editing site in the tyrosinase gene. Primers 7 to 58 were used for quantifying the mutations rates at the 13 off-target loci in the genome. Primers 59 to 62 were used for RT-qPCR to verify the mRNA expression levels of wild-type tyrosinase in parental cells and tyrosinase knock-out cells in Fig. S4.
