# Supplementary material for: CRISPR-clear imaging of melanin-rich B16-derived solid tumors
Source: Commun Biol. 2023 Apr 4;6:370. doi: 10.1038/s42003-023-04614-7 (PMC10073193; doi:10.1038/s42003-023-04614-7)
Supplement: Supplementary file 2 — Description of Additional Supplementary Files [file 42003_2023_4614_MOESM2_ESM.pdf]

Description of Additional Supplementary Files

File Name: Supplementary Video 1.

Description: 3D video rendering of images from Figure 1d of melanin(–) B16 tumors. Blood vessels were imaged that had been labeled by i.v. injection of DyLight 649 in the tail vein prior to sacrifice and fixation, while B16 cells were imaged by their tdTomato fluorescence and their nuclei with DAPI. White represents nuclear DAPI staining, red represents tdTomato-expressing B16 cells, and purple represents vasculature straining.

File Name: Supplementary Video 2.

Description: 3D video rendering of a 6mm x 6mm of an intact melanin(–) B16 tumor. Major ticks represent 1 mm and minor ticks resent 100 µm. White represents nuclear DAPI staining, red represents tdTomato-expressing B16 cells, and purple represents vasculature staining.

File Name: Supplementary Video 3.

Description: 3D video rendering of image depicted in Figure 1g of melanin(–) B16 tumors . Nuclei are shown in white.

File Name: Supplementary Video 4.

Description: 3D video rendering of image from Figure 3c. The fluorescence image was digitally reconstructed for nanoparticles in B16-tumor-bearing mice. Nanoparticles were injected to mimic nanoparticle-based therapies within the tumor microenvironment in immunodeficient RAG1-/- mice. White represents nuclear DAPI staining, cyan represents tdTomato-expressing B16 cells, green dots represent nanoparticles and purple represents vasculature straining. In the digital reconstruction of the optical images, not only the nanoparticles themselves can be observed but also the subcellular localization of them in the vasculature in B16 melanin(–) tumors.

File Name: Supplementary Data 1

Description: Source data
