# Supplementary material for: CRISPR-clear imaging of melanin-rich B16-derived solid tumors
Source: Commun Biol. 2023 Apr 4;6:370. doi: 10.1038/s42003-023-04614-7 (PMC10073193; doi:10.1038/s42003-023-04614-7)
Supplement: Supplementary file 8 — Reporting Summary [file 42003_2023_4614_MOESM8_ESM.pdf]

nature research

Corresponding author(s): Rajib Schubert, Andreas Plückthun and Junho K. Hur

Last updated by author(s): Feb 5, 2023

Reporting Summary

Nature Research wishes to improve the reproducibility of the work that we publish. This form provides structure for consistency and transparency in reporting. For further information on Nature Research policies, see our Editorial Policies and the Editorial Policy Checklist.

Statistics

For all statistical analyses, confirm that the following items are present in the figure legend, table legend, main text, or Methods section.

n/a	Confirmed	
☐	☒	The exact sample size (n) for each experimental group/condition, given as a discrete number and unit of measurement
☐	☒	A statement on whether measurements were taken from distinct samples or whether the same sample was measured repeatedly
☐	☒	The statistical test(s) used AND whether they are one- or two-sided<br>*Only common tests should be described solely by name; describe more complex techniques in the Methods section.*
☐	☒	A description of all covariates tested
☒	☐	A description of any assumptions or corrections, such as tests of normality and adjustment for multiple comparisons
☐	☒	A full description of the statistical parameters including central tendency (e.g. means) or other basic estimates (e.g. regression coefficient) AND variation (e.g. standard deviation) or associated estimates of uncertainty (e.g. confidence intervals)
☐	☒	For null hypothesis testing, the test statistic (e.g. F, t, r) with confidence intervals, effect sizes, degrees of freedom and P value noted<br>*Give P values as exact values whenever suitable.*
☒	☐	For Bayesian analysis, information on the choice of priors and Markov chain Monte Carlo settings
☒	☐	For hierarchical and complex designs, identification of the appropriate level for tests and full reporting of outcomes
☒	☐	Estimates of effect sizes (e.g. Cohen's d, Pearson's r), indicating how they were calculated

Our web collection on statistics for biologists contains articles on many of the points above.

Software and code

Policy information about availability of computer code

Data collection	3D image visualization, image quantification and image reconstructions were performed using Imaris version 9.5 imaging software (Bitplane)
Data analysis	Fluorescence measurements were analyzed using the open source software FIJI. Statistical analysis was performed using Prism (version 6.0b for Mac OSX; GraphPad Software).

For manuscripts utilizing custom algorithms or software that are central to the research but not yet described in published literature, software must be made available to editors and reviewers. We strongly encourage code deposition in a community repository (e.g. GitHub). See the Nature Research guidelines for submitting code & software for further information.

Data

Policy information about availability of data

All manuscripts must include a data availability statement. This statement should provide the following information, where applicable:

- Accession codes, unique identifiers, or web links for publicly available datasets
- A list of figures that have associated raw data
- A description of any restrictions on data availability

Provide your data availability statement here.

Field-specific reporting

Please select the one below that is the best fit for your research. If you are not sure, read the appropriate sections before making your selection.

☒ Life sciences ☐ Behavioural & social sciences ☐ Ecological, evolutionary & environmental sciences

For a reference copy of the document with all sections, see nature.com/documents/nr-reporting-summary-flat.pdf

Life sciences study design

All studies must disclose on these points even when the disclosure is negative.

Sample size	The sample size of n=9 mice (by strain) was determined by pilot experiments.
Data exclusions	Only tissues which failed to pass perfusion-fixation quality control were omitted from data analyses.
Replication	Animal experiments were done over 2-3 cohorts.
Randomization	Mice were randomly assigned to groups.
Blinding	Blinding was not necessary as no treatment was applied.

Reporting for specific materials, systems and methods

We require information from authors about some types of materials, experimental systems and methods used in many studies. Here, indicate whether each material, system or method listed is relevant to your study. If you are not sure if a list item applies to your research, read the appropriate section before selecting a response.

Materials & experimental systems

n/a	Involved in the study	
☐	☒	Antibodies
☐	☒	Eukaryotic cell lines
☒	☐	Palaeontology and archaeology
☐	☒	Animals and other organisms
☒	☐	Human research participants
☒	☐	Clinical data
☒	☐	Dual use research of concern

Methods

n/a	Involved in the study	
☒	☐	ChIP-seq
☐	☒	Flow cytometry
☒	☐	MRI-based neuroimaging

Antibodies

Antibodies used	All antibodies used are detailed in the Materials and Methods section of the paper.
Validation	Controls were used in each staining experiments as validation.

Eukaryotic cell lines

Policy information about cell lines

Cell line source(s)	All cell lines used are detailed in the Materials and Methods section of the paper.
Authentication	None of the cell lines were authenticated
Mycoplasma contamination	All cells tested negative from mycoplasma contamination.
Commonly misidentified lines (See ICLAC register)	-

Animals and other organisms

Policy information about studies involving animals; ARRIVE guidelines recommended for reporting animal research

Laboratory animals	All mice used are detailed in the Materials and Methods section of the paper.

Wild animals | -

Field-collected samples | -

Ethics oversight | Approved by the Cantonal Veterinary Office (Zurich, Switzerland) permit Nr. 237/2017,

Note that full information on the approval of the study protocol must also be provided in the manuscript.

Flow Cytometry

Plots

Confirm that:

- [x] The axis labels state the marker and fluorochrome used (e.g. CD4-FITC).
- [x] The axis scales are clearly visible. Include numbers along axes only for bottom left plot of group (a 'group' is an analysis of identical markers).
- [ ] All plots are contour plots with outliers or pseudocolor plots.
- [ ] A numerical value for number of cells or percentage (with statistics) is provided.

Methodology

Sample preparation	Detailed in the Materials and Methods section of the paper.
Instrument	LSRII Fortessa flow cytometer using a high-throughput sampler and FACSDiva software (Becton Dickinson)
Software	FCS Express 5 (De Novo Software)
Cell population abundance	-
Gating strategy	In figure S1, gates are set to the live cell population. In figure S6a, the live cell gate is shown on the entire population.

- [x] Tick this box to confirm that a figure exemplifying the gating strategy is provided in the Supplementary Information.
